# Comparative efficacy and safety of subcutaneous infliximab and vedolizumab in patients with Crohn’s disease and ulcerative colitis included in randomised controlled trials

**DOI:** 10.1186/s12876-024-03163-5

**Published:** 2024-03-27

**Authors:** Laurent Peyrin‐Biroulet, Perttu Arkkila, Alessandro Armuzzi, Silvio Danese, Marc Ferrante, Jordi Guardiola, Jørgen Jahnsen, Edouard Louis, Milan Lukáš, Walter Reinisch, Xavier Roblin, Philip J Smith, Taek Kwon, Jeeyoung Kim, Sangwook Yoon, Dong-Hyeon Kim, Raja Atreya

**Affiliations:** 1 Department of Gastroenterology, Centre Hospitalier Régional Universitaire de Nancy, Nancy, France; 2 Department of Gastroenterology, Helsinki University Hospital, Helsinki, Finland; 3 IBD Center, Humanitas Research Hospital, Rozzano, Milan, Italy; 4 Gastroenterology and Endoscopy, University Vita‐Salute San Raffaele, Milan, Italy; 5 Department of Gastroenterology and Hepatology, University Hospitals Leuven, KU Leuven, Leuven, Belgium; 6 Department of Digestive Diseases, Bellvitge University Hospital, Bellvitge Biomedical Research Institute‐IDIBELL, University of Barcelona, L’Hospitalet de Llobregat, Barcelona, Spain; 7 Institute of Clinical Medicine, University of Oslo, Oslo, Norway; 8 Department of Gastroenterology, University Hospital CHU of Liège, Liège, Belgium; 9 Clinical and Research Center for Inflammatory Bowel Diseases, ISCARE Clinical Centre, Prague, Czech Republic; 10 Department of Internal Medicine III, Medical University of Vienna, Vienna, Austria; 11 Department of Gastroenterology and Hepatology, University Hospital of Saint‐Etienne, Saint‐Etienne, France; 12 Department of Gastroenterology, Royal Liverpool Hospital, Liverpool University Hospitals NHS Foundation Trust, Liverpool, UK; 13 Celltrion, Incheon, Republic of Korea; 14 Medical Department 1, University Hospital Erlangen, Friedrich-Alexander-University of Erlangen-Nürnberg, Erlangen, Germany

**Keywords:** Biobetter, Bioinnovative, Inflammatory bowel disease, Subcutaneous infliximab, Tumour necrosis factor-α inhibitors, Vedolizumab

## Abstract

**Background:**

While indirect comparison of infliximab (IFX) and vedolizumab (VDZ) in adults with Crohn’s disease (CD) or ulcerative colitis (UC) shows that IFX has better effectiveness during induction, and comparable efficacy during maintenance treatment, comparative data specific to subcutaneous (SC) IFX (i.e., CT-P13 SC) *versus* VDZ are limited.

**Aim:**

Pooled analysis of randomised studies to compare efficacy and safety with IFX SC and VDZ in moderate-to-severe inflammatory bowel disease.

**Methods:**

Parallel-group, randomised studies evaluating IFX SC and VDZ in patients with moderate-to-severe CD or UC were identified. Eligible studies reported ≥ 1 prespecified outcome of interest at Week 6 (reflecting treatment during the induction phase) and/or at 1 year (Weeks 50-54; reflecting treatment during the maintenance phase). Prespecified efficacy and safety outcomes considered in this pooled analysis included the proportions of patients achieving disease-specific clinical responses, clinical remission, or discontinuing due to lack of efficacy, and the proportions of patients experiencing adverse events (AEs), serious AEs, infections, serious infections, or discontinuing due to AEs. Data from multiple studies or study arms were extracted and pooled using a random-effect model; comparative analyses were performed separately for patients with CD and UC.

**Results:**

We identified three eligible CD trials and four eligible UC trials that assigned over 1200 participants per disease cohort to either IFX SC or VDZ. In patients with CD, intravenous induction therapy with IFX demonstrated better efficacy (non-overlapping 95% confidence intervals [CIs]) compared with VDZ; during the maintenance phase, IFX SC showed numerically better efficacy (overlapping 95% CIs) than VDZ. A lower proportion of IFX SC-treated patients discontinued therapy due to lack of efficacy over 1 year. In patients with UC, efficacy profiles were similar with IFX SC and VDZ during the induction and maintenance phases, and a lower proportion of IFX SC-treated patients discontinued therapy due to lack of efficacy over 1 year. In both cohorts, safety profiles for IFX SC and VDZ were generally comparable during 1 year.

**Conclusion:**

IFX SC demonstrated better efficacy than VDZ in patients with CD, and similar efficacy to VDZ in patients with UC; 1-year safety was comparable with IFX SC and VDZ.

**Supplementary Information:**

The online version contains supplementary material available at 10.1186/s12876-024-03163-5.

## Background

Inflammatory bowel diseases (IBD) encompass a heterogeneous group of disorders, where the underlying pathology is chronic inflammation of the gastrointestinal (GI) tract [1–3]. Crohn’s disease (CD) and ulcerative colitis (UC) are the principal phenotypes of IBD, affecting multiple GI sites and the colon, respectively [1–3]. While most patients with CD or UC have mild-to-moderate disease, approximately 10%-20% experience a more aggressive disease course in the short term, which may require treatment with a biologic [1, 3–7]. European Crohn’s and Colitis Organisation (ECCO) guidelines for CD recommend the use of tumour necrosis factor-α inhibitors (TNFis; infliximab [IFX], adalimumab, or certolizumab pegol), vedolizumab (VDZ), or ustekinumab in patients with moderate-to-severe disease who have not responded to conventional therapy [6]. ECCO guidelines for UC recommend the use of TNFis (IFX, adalimumab, or golimumab), VDZ, ustekinumab, or tofacitinib in patients with moderate-to-severe disease who have an inadequate response or intolerance to conventional therapy [7]. Both guidelines recommend that the effective biologic used for induction of remission is continued for maintenance of remission [6, 7].

Considering IFX and VDZ in particular, ECCO treatment guidelines give a ‘strong’ level of recommendation for both options for the treatment of moderate-to-severe CD or UC [6, 7]. In the context of therapeutics not differentially recommended by treatment guidelines, other factors should be considered when making treatment decisions: for example, the British Society of Gastroenterology recommends that patient preference, cost, safety, likely adherence, and speed of response to the drug should be evaluated at the individual patient level when selecting between TNFis, VDZ, and ustekinumab for the treatment of IBD [3]. In the context of the coronavirus disease 2019 (COVID-19) pandemic, access to local infusion facilities and nosocomial infection risk have also been relevant considerations for treatment selection [8]. To date, there are no head-to-head studies directly comparing IFX and VDZ for the treatment of IBD, and limited comparative data are available from real-world settings [9]. For example, an ambidirectional cohort study of adult patients with UC conducted in a tertiary setting in the USA found similar rates of clinical response with IFX and VDZ [10]. Evidence from two recent systematic reviews and meta-analyses – the gold standard for evidence synthesis – also supports the use of IFX as a first-line agent for the treatment of moderate-to-severe CD or UC [9, 11].

IFX was originally formulated to be administered by intravenous (IV) infusion; however, a subcutaneous (SC) formulation of IFX, CT-P13 SC, received European Medicines Agency approval in July 2020 for the treatment of CD and UC, and is currently the only approved SC formulation of IFX [12–14]. Potential benefits of IFX SC for treating patients with CD or UC, *versus* IFX IV, include cost savings, increased convenience (*i.e.,* self-administration at home) and potentially reduced nosocomial exposure to severe acute respiratory syndrome coronavirus 2 [8, 15]. Notably, IFX SC has been cited as a candidate biobetter in an international Delphi consensus statement [16], based on the improved pharmacokinetic parameters associated with IFX SC relative to IFX IV, as observed in patients with IBD and rheumatoid arthritis (RA) [17, 18]. These differences in pharmacokinetic parameters between formulations may potentially translate to better efficacy with IFX SC over IFX IV, as suggested by findings from a phase I/III study in patients with RA [17].

In a previous systematic review and meta-analysis, we performed an indirect comparison of IFX and VDZ trials (both including IV and SC formulations) in adults with moderate-to severe CD or UC; we showed that IFX had better efficacy in the induction phase and comparable efficacy during the maintenance phase, and a similar overall safety profile, compared with VDZ [9]. Given the potential benefits of SC over IV dosing observed in patients with RA, we conducted a comparative analysis of efficacy and safety outcomes with IFX SC (CT-P13 SC) and VDZ (both IV and SC formulations) in patients with moderate-to-severe CD or UC.

## Methods

### Criteria for inclusion of studies in the present analyses

The current analysis was conducted using data from studies identified in a previously published systematic review and meta-analysis [9], which was based on a prospectively registered study protocol (PROSPERO number CRD42021177954). In brief, electronic searches of PubMed, Embase, and the Cochrane Library were conducted to identify study publications between 1 January 2010 and 30 April 2021 [9], and studies were selected based on title, abstract, and full-text screening, as previously described [9]. 

Studies included in the current analysis were parallel-group, randomised controlled trials (RCTs) that evaluated treatment with IFX SC, following induction therapy with IFX IV, or treatment with VDZ (either with VDZ IV or with VDZ SC [following IV induction therapy]). Studies reported one or more of the prespecified outcomes of interest at Week 6 (induction) and/or at 1 year (Weeks 50-54; maintenance phase).

Data were analysed separately for two cohorts of patients comprising adults (aged ≥ 18 years) with moderate-to-severe CD, and adults with moderate-to-severe UC. For the CD cohort, efficacy outcomes of interest were the proportions of patients achieving a ≥ 70-point decrease in Crohn’s Disease Activity Index (CDAI-70 response), a ≥ 100-point decrease in CDAI (CDAI-100 response), and clinical remission (absolute Crohn’s Disease Activity Index [CDAI] score < 150 points), at Week 6 (induction phase) and 1 year (Week 50-54; maintenance phase). For the UC cohort, efficacy outcomes of interest were the proportions of patients achieving a clinical response (defined as either a decrease from baseline in total Mayo score of ≥ 3 points and ≥ 30%, with an accompanying decrease of ≥ 1 point in the subscore for rectal bleeding or an absolute subscore for rectal bleeding of 0 or 1; or as a decrease from baseline in partial Mayo score of ≥ 2 points, with an accompanying decrease of ≥ 1 point in the subscore for rectal bleeding or an absolute subscore for rectal bleeding of 0 or 1), clinical remission (either total Mayo score of ≤ 2 points, with no individual subscore > 1 point, or partial Mayo score of ≤ 1 point), and mucosal healing (defined as absolute Mayo subscore of 0 or 1), during the induction and maintenance phases. The proportion of patients who discontinued due to a lack of efficacy during a 1-year period was an outcome of interest for both cohorts. Outcomes of interest relating to safety (both cohorts) were the proportions of patients experiencing adverse events (AEs), serious adverse events (SAEs), infections, or serious infections, and of those who discontinued due to AEs.

### Exploratory analysis

Given the literature search end date defined in the original protocol (30 April 2021), exploratory analyses were conducted to integrate findings from more recently published studies (to 30 November 2023) that otherwise met the eligibility criteria. Exploratory analyses were conducted to evaluate efficacy and safety outcomes of IFX SC and VDZ in patients with Crohn’s disease.

### Statistics

Outcome data were extracted from study reports using Microsoft Excel (Microsoft Corp., Redmond, WA, USA), as previously described [9]. For each outcome, data were pooled using a random-effect model, as previously described [9]. Comparative pooled analyses were only performed where the characteristics of the contributing studies were similar (*e.g.,* in terms of study population). All statistical analyses were performed using R (version 4.1.1; R Foundation for Statistical Computing, Vienna, Austria). 

## Results

### Search results

Preferred Reporting Items for Systematic Reviews and Meta-Analyses (PRISMA) flow diagrams for the original systematic review were published previously [9]. Three RCTs met the eligibility criteria for studies enrolling patients with CD and were included in the present analysis, as follows:

- ◦ IFX SC (one study): CT-P13 SC trial (NCT02883452) [19, 20].
- ◦ VDZ (two studies): GEMINI II (NCT00783692) [21–24] and GEMINI III (NCT01224171) [22, 23, 25].

Four RCTs met the eligibility criteria for studies enrolling patients with UC and were included in the present analysis:

- ◦ IFX SC (one study): CT-P13 SC trial (NCT02883452) [19, 20].
- ◦ VDZ (three studies): GEMINI I (NCT00783718) [26–30], VARSITY (NCT02497469) [31], and VISIBLE 1 (NCT02611830) [32].

For both cohorts (CD and UC), the other IFX studies included in the previous meta-analysis [9] did not meet the eligibility criteria for the current analysis, since treatment was with IFX IV only. For the CT-P13 SC trial, clinical study report data were also included in this analysis [20].

### Study characteristics

Characteristics of the included studies were previously reported [9].

#### Studies contributing to the CD analyses

**Fig. 1:**
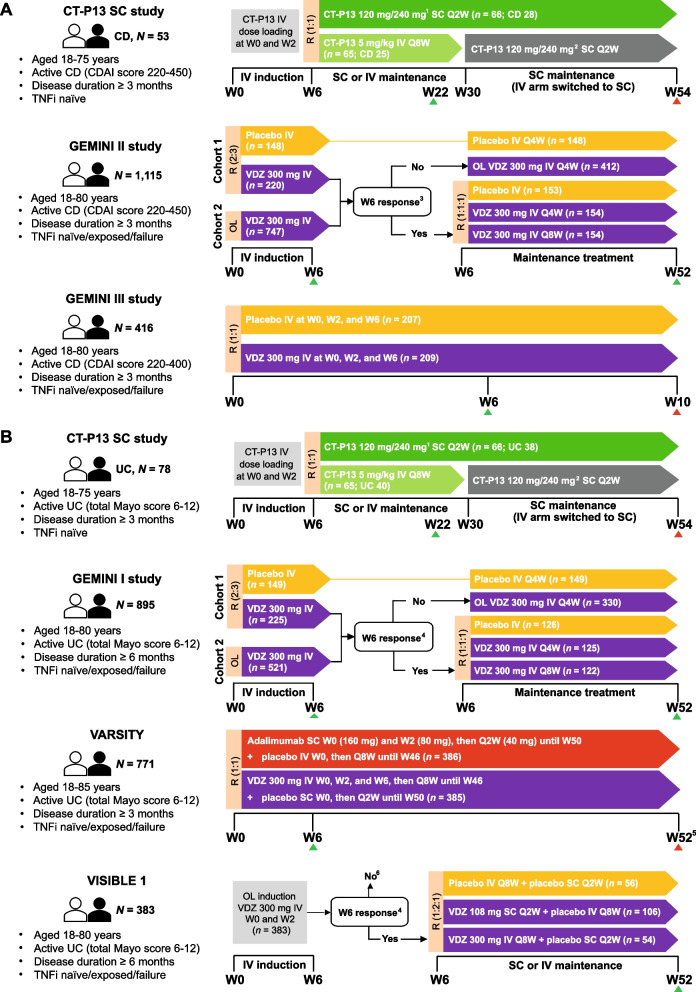
Summary of study designs for the included studies contributing data to the CD (A) and UC (B) analyses. ^1^For patients with W6 body weight < 80 kg or ≥ 80 kg, respectively. ^2^For patients with W30 body weight < 80 kg or ≥ 80 kg, respectively. ^3^Response defined as a ≥ 70-point reduction from baseline in CDAI score. ^4^Response was defined as a reduction in total Mayo score of ≥ 3 points and ≥ 30% from baseline, with an accompanying decrease in the rectal bleeding subscore of ≥ 1 point or absolute rectal bleeding subscore of ≤ 1. ^5^Final safety follow-up at W68. ^6^Patients without a clinical response at W6 received a third open-label dose of VDZ 300 mg IV and were reassessed for clinical response (see footnote 3) at W14; those achieving a clinical response had the option to enrol in an open-label extension study, and those who did not have a response were discontinued from the study. Green and red triangles indicate timing of primary and secondary endpoint assessments, respectively. CD: Crohn’s disease; CDAI: Crohn’s Disease Activity Index; IV: Intravenous; OL: Open-label; Q: Every; R: Randomisation; SC: Subcutaneous; TNFi: Tumour necrosis factor-α inhibitor; UC: Ulcerative colitis; VDZ: Vedolizumab; W: Week

The design of the three studies contributing data to the CD analysis are summarised in Fig. 1A. All studies were multinational, randomised trials [19, 21, 25]. Two of the studies included a double-blind period (GEMINI II [21] and GEMINI III [25]) and one study employed an open-label design (CT-P13 SC trial [19]). In the CT-P13 SC trial, all patients received CT-P13 5 mg/kg IV at Weeks 0 and 2, and patients were randomised to receive CT-P13 SC or CT-P13 IV at Week 6 [19]. During maintenance treatment (*i.e.,* Weeks 6-54), patients in the CT-P13 SC arm received CT-P13 120 mg (< 80 kg) or 240 mg (≥ 80 kg) SC every 2 weeks (Q2W); patients in the CT-P13 IV arm received CT-P13 5 mg/kg IV every 8 weeks (Q8W) from Weeks 6 to 22, and from Week 30 were switched to receive CT-P13 120/240 mg SC Q2W until Week 54 [19]. In GEMINI II, patients in the VDZ arms received VDZ 300 mg IV at Weeks 0 and 2, and those who had a clinical response at Week 6 were randomly assigned to receive VDZ 300 mg IV Q8W, VDZ 300 mg IV every 4 weeks (Q4W), or placebo, for up to 52 weeks [21]. The GEMINI III study specifically examined VDZ IV for induction of a clinical response; patients were randomised to receive VDZ 300 mg IV or placebo at Weeks 0, 2, and 6 [25]. Key endpoint assessments were performed up to Week 10, and patients without unacceptable AEs and who did not require surgery for CD during the study were eligible for the long-term open-label extension [25].

Across studies, adult patients (aged ≥ 18 years) were required to have a diagnosis of CD and a disease duration of ≥ 3 months prior to first administration of study drug [19, 21, 25]. Patients enrolled in the CT-P13 SC trial were required to be TNFi naïve and not to have received any prior biologic for the treatment of CD [19]. In contrast, eligibility criteria for the GEMINI II and GEMINI III studies required patients to have had inadequate/no response to, or unacceptable side effects with, corticosteroids/glucocorticoids, immunosuppressants, or TNFis [21, 25]. The primary efficacy analysis of the GEMINI III study was restricted to patients with prior TNFi failure [25]. Patients in GEMINI II and GEMINI III were not permitted to have received previous treatment with VDZ, natalizumab, efalizumab, or rituximab [21, 25].

A total of 1229 participants were initially assigned to the relevant treatment arms (IFX, *n* = 53; VDZ, *n* = 1176) of the selected studies. Baseline characteristics (*i.e.,* age, sex, and body weight) were generally consistent across the relevant arms (Supplementary Table 1). Patients in the CT-P13 SC trial had a mean disease duration of 4.5 years, compared with a median of 8.4 years in GEMINI II and a mean of 9.2 years in GEMINI III [19–21, 25]. Approximately 36% of patients in the CT-P13 SC trial (with either CD or UC) were receiving concomitant corticosteroids, compared with approximately 52% and 53% of VDZ-treated patients in GEMINI II and GEMINI III, respectively [19, 21, 25]. Reflecting the inclusion criteria of the respective studies, all patients enrolled in the CT-P13 SC trial were TNFi naïve, whereas 64% of patients had previously received TNFi therapy for CD in the VDZ arms of GEMINI II (60% had prior TNFi failure), and 76% of patients had previously failed ≥ 1 TNFi in the VDZ arm of GEMINI III [19, 21, 25]. 

#### Studies contributing to the UC analyses

The design of the four studies contributing data to the UC analysis are summarised in Fig. 1B. The studies were all multinational, randomised trials with a duration of ≥ 52 weeks [19, 26, 31, 32]. Three of the studies included a double-blind period (GEMINI I, VARSITY, and VISIBLE 1) [26, 31, 32] and one study had an open-label design (CT-P13 SC trial) [19]. The IFX regimens used in the CT-P13 SC trial are detailed in the previous section. In the GEMINI I, VARSITY, and VISIBLE 1 studies, patients assigned to the VDZ arms initially received VDZ 300 mg IV at Weeks 0 and 2 for induction [26, 31, 32]. In GEMINI I, patients with a clinical response at Week 6 were subsequently randomly assigned to receive VDZ 300 mg IV Q8W, VDZ 300 mg IV Q4W, or placebo, for up to 52 weeks [26]. In the VARSITY study, patients who were initially randomised to receive VDZ continued to receive VDZ 300 mg IV Q8W during the maintenance period (Weeks 6-46) [31]. The VISIBLE 1 study was the only VDZ trial to evaluate outcomes with VDZ SC treatment: in this study, patients with a clinical response at Week 6 were randomised to receive either VDZ 300 mg IV Q8W, VDZ 108 mg SC Q2W, or placebo [32]. 

Across studies, adult patients (aged ≥ 18 years) were required to have a diagnosis of UC, with disease duration of either ≥ 6 months prior to enrolment (GEMINI I and VISIBLE 1) or ≥ 3 months prior to screening/administration of study drug (VARSITY and CT-P13 SC, respectively) [19, 26, 31, 32]. Patients enrolled in the CT-P13 SC trial were required to be TNFi naïve and not to have received any prior biologics for the treatment of UC [19]. Eligibility criteria for the GEMINI I study required prior treatment failure with either glucocorticoids, immunosuppressants, or TNFis [26]. Patients were ineligible if they had received TNFis within 60 days of enrolment, and they were also not permitted to have previously received VDZ, natalizumab, efalizumab, or rituximab [26]. In VARSITY, patients who were TNF naïve and had no response or loss of response to conventional treatments were eligible, as were patients with prior TNFi failure (excluding adalimumab); the latter group was capped at 25% of the study population [31]. Patients were ineligible if they had previously received any approved biologic within 60 days (or 5 half-lives) prior to screening, or if they had previously received VDZ, natalizumab, efalizumab, adalimumab, etrolizumab, AMG-181, anti-mucosal addressin cell adhesion molecule-1 (anti-MAdCAM-1) antibodies, or rituximab [31]. Patients in VISIBLE 1 were required to have had an inadequate response, loss of response, or intolerance to ≥ 1 other treatment (corticosteroid, immunomodulator, or TNFi) [32]. Prior exposure to any biologic was not permitted within 60 days or 5 half-lives of screening [32]. Previous exposure to any anti-integrin therapy (including VDZ, natalizumab, efalizumab, etrolizumab, or AMG-181), anti-MAdCAM-1 antibodies, or rituximab was also not permitted [32].

A total of 1369 participants (IFX, *n* = 78; VDZ, *n* = 1291) were initially assigned to the relevant treatment arms of the selected studies. Baseline characteristics (*i.e.,* age, sex, and body weight) were generally consistent across the selected studies (Supplementary Table 2). Patients in the CT-P13 SC trial had a mean disease duration of 6.6 years compared with a mean duration of 6.8-8.2 years across the VDZ arms of the GEMINI I, VARSITY, and VISIBLE 1 studies [19, 20, 26, 31, 32]. Approximately 36% of patients in the CT-P13 SC trial (with either CD or UC) were receiving concomitant corticosteroids, compared with approximately 36%-53% of VDZ-treated patients across the GEMINI I, VARSITY, and VISIBLE 1 studies [19, 26, 31, 32]. Reflecting the inclusion criteria of the respective studies, all patients enrolled in the CT-P13 SC trial were TNFi naïve, whereas 40.8% and 18.7% of patients had prior failure of TNFi therapy in the VDZ arms of GEMINI I and VARSITY, respectively [19, 26, 31]. Prior use of TNFi therapy was reported in 37.7% and 44.4% of patients in the VDZ SC and VDZ IV arms of the VISIBLE 1 study, respectively [32].

### Risk of bias and generalisability of the included studies

A quality assessment for the included studies was previously reported [9] and is briefly summarised below. Across the 21 assessments performed for studies contributing to the CD analyses, 13 assessments were considered to be at low risk of bias and 8 were considered to be at high risk of bias. The CT-P13 SC trial was considered to be at high risk of bias for three domains owing to the open-label study design and because the results were not reported separately for the CD and UC populations. The GEMINI II study was determined to be at high risk of bias for four domains. The high risk of ‘other’ bias identified was applicable to the maintenance phase only, because of the inclusion of induction responders only in the maintenance phase. The GEMINI III study was considered to be at high risk of bias for blinding of outcome assessments.

Across the 28 assessments performed for studies contributing to the UC analyses, 18 assessments were considered to be at low risk of bias, 9 were considered to be at high risk of bias, and 1 assessment was considered to be at unclear risk of bias. The risk of bias associated with the CT-P13 SC trial is as described for the CD analyses. The GEMINI I study was considered to be at high risk of bias for allocation concealment, blinding of participants and personnel, and ‘other’ bias, and the risk of bias was unclear for blinding of outcome assessment. The VISIBLE 1 study was considered to be at high risk of bias for blinding of outcome assessment and of blinding of participants and personnel, and of ‘other’ bias. The high risk of ‘other’ bias in GEMINI I and VISIBLE 1 was due to the selective inclusion of patients who achieved a clinical response during induction in the subsequent maintenance phase (as above, this rating only applies to the maintenance phase data). 

### Comparative efficacy and safety in patients with CD

**Fig. 2:**
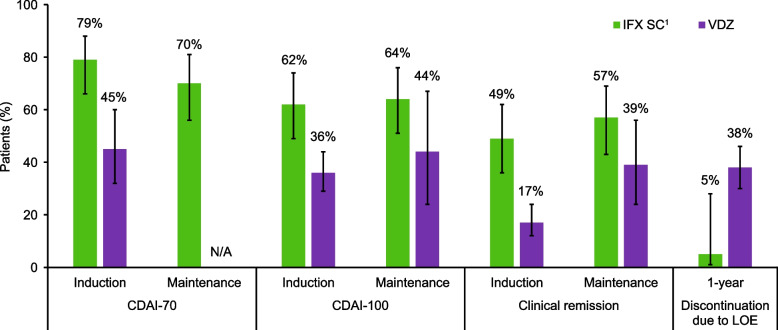
Comparison of IFX SC^1^ *versus* VDZ for key efficacy outcomes in patients with Crohn’s disease. ^1^Results from the induction period were analysed for patients included in the IFX SC group who had received IFX IV induction therapy. Error bars show 95% CIs. CDAI-100: ≥ 100-point decrease in Crohn’s Disease Activity Index; CDAI-70: ≥ 70-point decrease in Crohn’s Disease Activity Index; CI: Confidence interval; IFX: Infliximab; IV: Intravenous; LOE: Lack of efficacy; SC: Subcutaneous; VDZ: Vedolizumab

A summary of findings for the pooled analyses of data from patients with CD is presented in Fig. 2 and Supplementary Table 3 (efficacy) and Table 1 (safety).

**Table 1:** Comparative safety of IFX SC and VDZ during a 1-year period in patients with Crohn’s disease

**Outcome**	**Group**	**Events**	**Total**	**Proportion (95% CI)**	**Heterogeneity (*I***^**2**^**)**
AE	IFX SC	40	53	0.76 (0.58-0.88)	0%
	VDZ	788	985	0.78 (0.59-0.90)	98%
SAE	IFX SC	5	53	0.09 (0.04-0.21)	0%
	VDZ	198	985	0.16 (0.08-0.30)	93%
Infection	IFX SC	20	53	0.38 (0.26-0.51)	0%
	VDZ	73	427	0.17 (0.14-0.21)	24%
Serious infection	IFX SC	2	53	0.04 (0.01-0.14)	0%
	VDZ	44	985	0.04 (0.02-0.08)	67%
Discontinuation due to AEs	IFX SC	2	53	0.04 (0.01-0.14)	0%
	VDZ	89	985	0.07 (0.03-0.15)	84%

*AE* Adverse event, *CI* Confidence interval, *IFX* Infliximab, *SAE* Serious adverse event, *SC* Subcutaneous, *VDZ* Vedolizumab

#### Efficacy

For all efficacy outcomes during the induction phase, IFX IV induction therapy yielded better efficacy than with VDZ, with non-overlapping 95% CIs (CDAI-70: 79% *vs* 45%; CDAI-100: 62% *vs* 36%; clinical remission: 49% *vs* 17%) (Supplementary Table 3 and Fig. 2). 

During the maintenance phase, numerically higher proportions of patients achieved a CDAI-100 response and clinical remission in the IFX SC group than in the VDZ group (CDAI-100: 64% *vs* 44%; clinical remission: 57% *vs* 39% [Supplementary Table 3 and Fig. 2]); however, the 95% CIs were overlapping. 

A significantly lower proportion of patients discontinued during a 1-year period due to lack of efficacy in the IFX SC group (5% [95% CI: 1%-28%]) than in the VDZ group (38% [95% CI: 30%-46%]) (Supplementary Table 3 and Fig. 2).

#### Safety

Similar proportions of patients in the IFX SC and VDZ groups experienced AEs (76% *vs* 78%), SAEs (9% *vs* 16%), serious infections (4% *vs* 4%), and discontinuations due to AEs (4% *vs* 7%) during a 1-year period (Table 1); 95% CIs were overlapping for each comparison. During the same period, a significantly higher proportion of patients had an infection in the IFX SC group (38% [95% CI: 26%-51%]) compared with the VDZ group (17% [95% CI: 14%-21%]) (Table 1).

### Comparative efficacy and safety in patients with UC

**Fig. 3:**
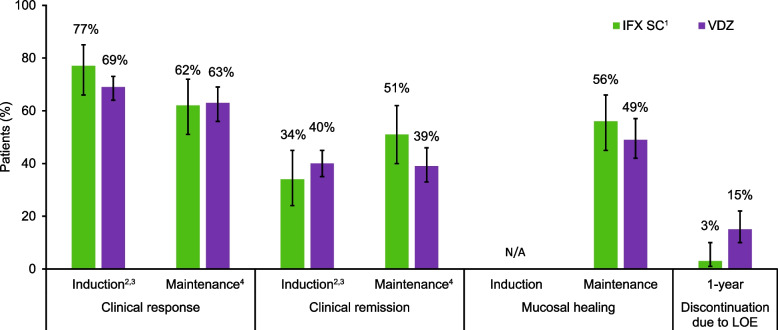
Comparison of IFX SC^1^ *versus* VDZ for key efficacy outcomes in patients with ulcerative colitis. ^1^Results from the induction period were analysed for patients included in the IFX SC group who had received IFX IV induction therapy. ^2^Evaluated based on partial Mayo score. ^3^Data for VDZ are based on data from a single arm of the VARSITY trial. ^4^Evaluated based on total Mayo score. Error bars show 95% CIs. CI: Confidence interval; IFX: Infliximab; IV: Intravenous; LOE: Lack of efficacy; SC: Subcutaneous; VDZ: Vedolizumab

A summary of findings for the pooled analyses of data from patients with UC is presented in Fig. 3 and Supplementary Table 4 (efficacy) and Table 2 (safety).

**Table 2:** Comparative safety of IFX SC and VDZ during a 1-year period in patients with ulcerative colitis

**Outcome**	**Group**	**Events**	**Total**	**Proportion (95% CI)**	**Heterogeneity (*I***^**2**^**)**
AE	IFX SC	52	78	0.67 (0.56-0.76)	0%
	VDZ	350	543	0.64 (0.60-0.68)	44%
SAE	IFX SC	9	78	0.12 (0.06-0.21)	0%
	VDZ	59	543	0.11 (0.09-0.14)	0%
Infection	IFX SC	24	78	0.31 (0.22-0.42)	0%
	VDZ	139	543	0.26 (0.22-0.29)	14%
Serious infection	IFX SC	5	78	0.06 (0.03-0.14)	35%
	VDZ	9	489	0.02 (0.01-0.03)	0%
Discontinuation due to AEs	IFX SC	2	78	0.03 (0.01-0.10)	0%
	VDZ	31	667	0.05 (0.03-0.07)	0%

*AE* Adverse event, *CI* Confidence interval, *IFX* Infliximab, *SAE* Serious adverse event, *SC* Subcutaneous, *VDZ* Vedolizumab

#### Efficacy

During the induction phase, the proportions of patients who achieved a clinical response or clinical remission based on partial Mayo score was similar with induction therapy with IFX IV or with VDZ, noting that data for VDZ were based on a single arm of the VARSITY trial, and a comparative analysis could not be performed (clinical response: 77% *vs* 69%; clinical remission: 34% *vs* 40%) (Supplementary Table 4 and Fig. 3). 

During the maintenance period, similar proportions of patients in the IFX SC and VDZ groups achieved a clinical response (62% *vs* 63%), clinical remission (51% *vs* 39%), and mucosal healing (56% *vs* 49%) (Supplementary Table 4 and Fig. 3). 

A numerically lower proportion of patients discontinued during a 1-year period due to lack of efficacy in the IFX SC group (3% [95% CI: 1%-10%]) than the VDZ group (15% [95% CI: 10%-22%]) (Supplementary Table 4).

#### Safety

Similar proportions of patients in the IFX SC and VDZ groups experienced AEs (67% *vs* 64%), SAEs (12% *vs* 11%), infections (31% *vs* 26%), and discontinuations due to AEs (3% *vs* 5%) during a 1-year period (Table 2); 95% CIs were overlapping for each comparison. A numerically higher proportion of patients experienced serious infections in the IFX SC group (6% [95% CI: 3%-14%]) compared with the VDZ group (2% [95% CI: 1%-3%]) (Table 2).

### Exploratory analysis

For the exploratory analyses, the only additional full publication identified was for VISIBLE 2, a multinational, randomised, double-blind, placebo-controlled trial that evaluated VDZ SC maintenance treatment in patients with moderate-to-severe CD [33]. In addition, data for LIBERTY-CD and LIBERTY-UC were identified from recently published congress abstracts; both are randomised, placebo-controlled trials of IFX SC in patients with CD or UC [34, 35]. 

In VISIBLE 2, patients received open-label VDZ IV 300 mg induction at Weeks 0 and 2, and Week 6 responders were randomised (2:1) to receive VDZ SC 108 mg Q2W or placebo from Weeks 6 to 50 [33]. Eligible patients were required to have a previous inadequate response or intolerance to corticosteroids, immunomodulators, and/or TNFis; 61% of the 275 patients randomised to VDZ SC had prior TNFi exposure [33]. In LIBERTY-CD and LIBERTY-UC, patients received open-label IFX SC 5mg/kg at Weeks 0, 2, and 6; at Week 10, clinical responders were randomised (2:1) to receive IFX SC 120 mg or placebo Q2W until Week 54 [34, 35]. In terms of baseline characteristics for the VDZ SC arm of the VISIBLE 2 study, mean (SD) age was 38.2 (13.9) years, body weight was 74.1 (19.0) kg, disease duration was 9.5 (8.3) years, and 43% of patients were female [33]. Baseline characteristics for the patients in the LIBERTY studies were not reported in the published congress abstracts [34, 35].

Following the addition of data from VISIBLE 2 and LIBERTY-CD (Supplementary Table 5), the proportions of patients achieving a CDAI-100 response and clinical remission remained numerically higher with IFX SC compared with VDZ during the maintenance phase (CDAI-100: 65% *vs* 48%; clinical remission: 61% *vs* 44%); the 95% CIs remained overlapping. The proportion of patients who discontinued due to lack of efficacy during a 1-year period was numerically lower in the IFX SC group than the VDZ group (5% *vs* 33%); however, the 95% CIs were overlapping.

Following the addition of data from LIBERTY-UC, the proportions of patients in the IFX SC and VDZ groups who achieved a clinical response (56% *vs* 63%) and clinical remission (45% *vs* 39%) during the maintenance period remained similar (Supplementary Table 6).

The proportions of patients in the IFX SC and VDZ groups who experienced AEs (76% *vs* 77%), SAEs (9% *vs* 14%), infections (38% *vs* 24%), and discontinuations due to AEs (4% *vs* 7%) during a 1-year period remained similar (Supplementary Table 7); 95% CIs were overlapping for each comparison.

## Discussion

The present analysis included data from six RCTs that evaluated either IFX SC or VDZ (IV or SC) in adults with CD or UC [19, 21, 22, 26, 31, 32]; the RCTs were identified through the previously published systematic review and meta-analysis [9], with the addition of unpublished clinical study report data provided by the study sponsor for the CT-P13 SC trial (NCT02883452) [20]. In contrast to the previously published systematic review and meta-analysis [9] extended by the current analysis, the interventions of interest herein were IFX SC (i.e., CT-P13 SC), specifically, and VDZ. In patients with CD, IFX IV induction therapy was associated with better efficacy outcomes compared with VDZ, while during the maintenance phase, IFX SC showed numerically better efficacy than VDZ. Safety profiles for IFX SC and VDZ were generally comparable during a 1-year period. Although higher rates of infection occurred with IFX SC than with VDZ, rates of serious infection were comparable between treatments. In patients with UC, efficacy profiles with IFX IV induction therapy and with IFX SC during the maintenance phase were similar to corresponding findings with VDZ (IV or SC). Safety profiles were also generally comparable for IFX SC and VDZ treatment during the 1-year period.

All the included studies enrolled adult patients with moderate-to-severe CD or UC; thus, the findings are generalisable to these populations. Risk of bias associated with the included studies was principally judged to be low; however, some aspects of the study designs were rated as being at high risk for bias. Notably, the GEMINI I, GEMINI II, and VISIBLE 1 trials were rated as being at high risk of bias for the category ‘other’ bias, because only patients who achieved a clinical response during induction went on to participate in the maintenance phase, which could potentially lead to a higher estimate of efficacy during the maintenance phase than if patients who did not achieve a clinical response were also included.

In terms of first-line biologic selection for patients with moderate-to-severe CD or UC who have not responded or are intolerant to conventional therapy, the previous systematic review and meta-analysis that included data from both IFX IV and IFX SC studies showed that IFX had better efficacy in the induction phase and comparable efficacy during the maintenance phase, compared with VDZ, in patients with CD or UC [9]. Building on this, the present findings suggest that IFX SC has better efficacy than VDZ in patients with CD, and similar efficacy to VDZ in patients with UC. Similarly, just as safety profiles with IFX (IV or SC) and VDZ were shown to be comparable in the previous review [9], findings for safety outcomes were comparable between IFX SC and VDZ in the present analysis. Our findings also align with those of a systematic review and meta-analysis conducted by Singh and colleagues, which suggested that either IFX (combined with azathioprine) or adalimumab might be the preferred choice for first-line therapy for induction of clinical remission in patients with moderate-to-severe CD [11].

Our findings should be interpreted cautiously given differences in the treatment background of patients included in the IFX and VDZ trials: all VDZ studies permitted enrolment of patients with prior TNFi failure, accounting for 47.5% of VDZ-treated patients overall [21, 25, 26, 31, 32], while the CT-P13 SC trial only enrolled patients who had received no prior biologics for UC or CD [19]. It was not possible to perform a sub-group analysis in biologic-naïve patients, as relevant data were unavailable for VDZ studies. Interpretation of our findings should also be mindful that SC treatment with either IFX (in the CT-P13 SC trial) or VDZ (in VISIBLE 1) followed IV-administered induction therapy [19, 32], consistent with the approved posology for CT-P13 SC and VDZ SC [12, 36]. Therefore, in SC treatment groups, findings from the induction period reflect the results of IV treatment. Additionally, it is important to note that patients in the IFX IV arm of the CT-P13 SC trial, who switched to receive IFX SC treatment from Week 30 onwards [19], were also included in the current analysis. Finally, compared with VDZ, a relatively small number of patients treated with IFX SC in the study conducted to date were available for inclusion in this analysis; however, two phase III studies in large cohorts of patients with moderate-to-severe CD (NCT03945019) and moderate-to-severe UC (NCT04205643) investigating the efficacy and safety of CT-P13 SC have recently been completed [37, 38]. In future, it would be valuable to update the comparative analysis to incorporate findings from these studies. Given the inclusion period, no studies evaluating VDZ SC treatment in patients with CD were included in the main analysis. We therefore conducted exploratory analyses with a later inclusion date, that incorporated findings from the VISIBLE 2, LIBERTY-CD, and LIBERTY UC studies [33–35]. Overall, results of the exploratory analyses were consistent with the main findings, suggesting no major impact of VDZ SC availability alongside VDZ IV for the treatment of CD on the evaluated efficacy and safety outcomes.

The present analysis has several strengths. Studies were selected from RCTs identified through a comprehensive electronic search strategy conducted as part of a recently published systematic review and meta-analysis [9], with published data for the CT-P13 SC trial supplemented with unpublished clinical study report data provided by the study sponsor [19, 20]. Thus, the analysis extends the previous meta-analysis findings through focusing on results for patients who received IFX SC treatment in the CT-P13 SC trial. 

In summary, our findings add to the evidence supporting IFX as a first-line biologic treatment option for patients with moderate-to severe CD or UC who have not responded, or are intolerant, to conventional therapy. In the context of the limited number of treatment options available for patients with IBD, our results suggest that IFX SC provides comparable or better efficacy than VDZ, with a similar safety profile. As highlighted by a recent review article, the flexibility afforded by SC *versus* IV dosing of biologics offers several potential benefits, including a reduced need for hospital visits in the era of COVID-19 and the convenience of at-home administration [8].

## Conclusion

In patients with moderate-to-severe CD, IFX IV induction therapy and IFX SC maintenance treatment were associated with potentially improved efficacy outcomes compared with VDZ, while efficacy findings were similar between IFX IV induction or IFX SC maintenance and VDZ in patients with moderate-to-severe UC. Safety profiles during a 1-year period were generally comparable between IFX SC and VDZ in both cohorts. Further evaluation is required to confirm these findings pending the availability of larger datasets for IFX SC.

### Supplementary Information

**Supplementary material 1.** 

## Data Availability

All data generated or analysed during this study are included in this published article.

## Abbreviations

AE: Adverse event
CD: Crohn’s disease
CDAI: Crohn’s Disease Activity Index
CDAI-70: ≥ 70-point decrease in Crohn’s Disease Activity Index
CDAI-100: ≥ 100‑point decrease in Crohn’s Disease Activity Index
CI: Confidence interval
COVID‑19: Coronavirus disease 2019
ECCO: European Crohn’s and Colitis Organisation
GI: Gastrointestinal
IBD: Inflammatory bowel disease
IFX: Infliximab
IV: Intravenous
LOE: Lack of efficacy
OL: Open-label
PRISMA: Preferred Reporting Items for Systematic Reviews and Meta-Analyses
Q: Every
R: Randomisation
RA: Rheumatoid arthritis
RCT: Randomised controlled trial
SAE: Serious adverse event
SC: Subcutaneous
TNFi: Tumour necrosis factor-α inhibitor
UC: Ulcerative colitis
VDZ: Vedolizumab
W: Week
