# Supplementary material for: Comparative efficacy and safety of subcutaneous infliximab and vedolizumab in patients with Crohn’s disease and ulcerative colitis included in randomised controlled trials
Source: BMC Gastroenterol. 2024 Mar 27;24:121. doi: 10.1186/s12876-024-03163-5 (PMC10967176; doi:10.1186/s12876-024-03163-5)
Supplement: Supplementary file 1 — Supplementary material 1. [file 12876_2024_3163_MOESM1_ESM.doc]

Comparative efficacy and safety of subcutaneous infliximab and vedolizumab in patients with Crohn’s disease and ulcerative colitis included in randomised controlled trials

Laurent Peyrin‐Biroulet, Perttu Arkkila, Alessandro Armuzzi, Silvio Danese, Marc Ferrante, Jordi Guardiola, Jørgen Jahnsen, Edouard Louis, Milan Lukáš, Walter Reinisch, Xavier Roblin, Philip J Smith, Taeksang Kwon, Jeeyoung Kim, Sangwook Yoon, Dong-Hyeon Kim, Raja Atreya

**Correspondence to:** Raja Atreya (raja.atreya@uk-erlangen.de)
Medical Department 1, University Hospital Erlangen, Friedrich-Alexander-University of Erlangen-Nürnberg, Ulmenweg 18, Erlangen, Germany

## Supplementary Table 1 Baseline characteristics of participants with Crohn’s disease, adapted from Peyrin-Biroulet *et al*. [[1](#_ENREF_1)] in accordance with the terms of the Creative Commons Attribution 4.0 International License (https://creativecommons.org/licenses/by/4.0/legalcode).

| **Randomised controlled trial** | **Randomised arms included in  meta-analyses** | ***N*** | **Age, median (range) years** | **Sex, % female** | **Body weight, median (range) kg** | **Disease duration, years** |
| --- | --- | --- | --- | --- | --- | --- |
| CT-P13 SC trial [[2](#_ENREF_2), [3](#_ENREF_3)] | CT-P13 SC  CT-P13 IV | 28  25 | 34.0 (18-69)  35.0 (19-53) | 43  56 | 66.0 (45.2-110.0)  64.4 (43.2-116.2) | 4.5 (6.5)^1^  5.6 (5.6)^1^ |
| GEMINI 2 [[4](#_ENREF_4)] | VDZ | 967 | 35.7 (11.9)^1^ | 53 | 69.9 (19.5)^1^ | 9.2 (7.8)^1^ |
| GEMINI 3 [[5](#_ENREF_5)] | VDZ | 209 | 36.9 (20-69) | 56 | 69.5 (40-144)^2^ | 8.4 (0.3-41.8)^3^ |

^1^Data are mean (standard deviation).

^2^Data are mean (range).

^3^Data are median (range).

IV: Intravenous; SC: Subcutaneous; VDZ: Vedolizumab.

## Supplementary Table 2 Baseline characteristics of participants with ulcerative colitis, adapted from Peyrin-Biroulet *et al*. [[1](#_ENREF_1)] in accordance with the terms of the Creative Commons Attribution 4.0 International License (https://creativecommons.org/licenses/by/4.0/legalcode).

| **Randomised controlled trial** | **Randomised arms included in  meta-analyses** | ***N*** | **Age, median (range) years** | **Sex, % female** | **Body weight, median (range) kg** | **Disease duration, years** |
| --- | --- | --- | --- | --- | --- | --- |
| CT-P13 SC trial [[2](#_ENREF_2), [3](#_ENREF_3)] | CT-P13 SC  CT-P13 IV | 38  40 | 33.0 (18-65)  37.0 (18-70) | 47  40 | 66.5 (47.2-117.0)  72.7 (50.0-115.3) | 6.6 (5.5)^1^  6.0 (6.7)^1^ |
| GEMINI 1 [[6](#_ENREF_6)] | VDZ | 746 | 40.1 (13.2)^1^ | 42 | 73.6 (18.7)^1^ | 6.8 (6.2)^1^ |
| VARSITY [[7](#_ENREF_7)] | VDZ | 385 | 40.8 (13.7)^1^ | 39 | 72.7 (17.0)^1^ | 7.3 (7.2)^1^ |
| VISIBLE 1 [[8](#_ENREF_8)] | VDZ SC  VDZ IV | 106  54 | 38.1 (13.1)  41.6 (14.1) | 39  43 | 71.6 (17.2)  77.0 (16.9) | 8.0 (6.2)^1^  8.2 (5.9)^1^ |

^1^Data are mean (standard deviation).

IV: Intravenous; SC: Subcutaneous; VDZ: Vedolizumab.

**Supplementary Table 3** **Comparative efficacy of IFX SC and VDZ in patients with Crohn’s disease.**

| **Treatment period** | **Outcome** | **Group** | **Events** | **Total** | **Proportion (95% CI)** | **Heterogeneity (*I*^2^)** |
| --- | --- | --- | --- | --- | --- | --- |
| Induction | CDAI-70 response | IFX SC^1^ | 42 | 53 | 0.79 (0.66-0.88) | – |
|  |  | VDZ | 98 | 214 | 0.45 (0.32-0.60) | 89% |
|  | CDAI-100 response | IFX SC^1^ | 33 | 53 | 0.62 (0.49-0.74) | – |
|  |  | VDZ | 153 | 423 | 0.36 (0.29-0.44) | 68% |
|  | Clinical remission | IFX SC^1^ | 26 | 53 | 0.49 (0.36-0.62) | – |
|  |  | VDZ | 70 | 423 | 0.17 (0.12-0.24) | 71% |
| Maintenance | CDAI-70 response | IFX SC | 37 | 53 | 0.70 (0.56-0.81) | – |
|  |  | VDZ | – | – | – | – |
|  | CDAI-100 response | IFX SC | 34 | 53 | 0.64 (0.51-0.76) | – |
|  |  | VDZ | 64 | 148 | 0.44 (0.24-0.67) | 93% |
|  | Clinical remission | IFX SC | 30 | 53 | 0.57 (0.43-0.69) | – |
|  |  | VDZ | 57 | 148 | 0.39 (0.24-0.56) | 88% |
| 1 year | Discontinuation due to lack of efficacy | IFX SC | 3 | 53 | 0.05 (0.01-0.28) | – |
|  |  | VDZ | 58 | 154 | 0.38 (0.30-0.46) | – |

^1^Results from the induction period were analysed for patients included in the IFX SC group who had received IFX IV induction therapy.

CDAI-100: ≥ 100-point decrease in Crohn’s Disease Activity Index; CDAI-70: ≥ 70-point decrease in Crohn’s Disease Activity Index; CI: Confidence interval; IFX: Infliximab; IV: Intravenous; SC: Subcutaneous; VDZ: Vedolizumab.

**Supplementary Table 4 Comparative efficacy of IFX SC and VDZ in patients with ulcerative colitis.**

| **Treatment period** | **Outcome** | **Group** | **Events** | **Total** | **Proportion (95% CI)** | **Heterogeneity (*I*^2^)** |
| --- | --- | --- | --- | --- | --- | --- |
| Induction | Clinical response^1^ | IFX SC^2^ | 59 | 77 | 0.77 (0.66-0.85) | – |
|  | Clinical remission^1^ | IFX SC^2^ | 26 | 77 | 0.34 (0.24-0.45) | – |
| Maintenance | Clinical response^3^ | IFX SC | 48 | 77 | 0.62 (0.51-0.72) | – |
|  |  | VDZ | 176 | 282 | 0.63 (0.56-0.69) | 51% |
|  | Clinical remission^3^ | IFX SC | 39 | 77 | 0.51 (0.40-0.62) | – |
|  |  | VDZ | 243 | 665 | 0.39 (0.33-0.46) | 73% |
|  | Mucosal healing | IFX SC | 43 | 77 | 0.56 (0.45-0.66) | – |
|  |  | VDZ | 304 | 665 | 0.49 (0.42-0.57) | 78% |
| 1 year | Discontinuation due to lack of efficacy | IFX SC | 2 | 78 | 0.03 (0.01-0.10) | – |
|  |  | VDZ | 96 | 667 | 0.15 (0.10-0.22) | 82% |

^1^Evaluated based on partial Mayo score.

^2^Results from the induction period were analysed for patients included in the IFX SC group who had received IFX IV induction therapy.

^3^Evaluated based on total Mayo score.

CI: Confidence interval; IFX: Infliximab; IV: Intravenous; SC: Subcutaneous; VDZ: Vedolizumab.

**Supplementary Table 5 Exploratory analysis for outcomes reported in the VISIBLE 2 study [**[**9**](#_ENREF_9)**] and LIBERTY-CD [**[**10**](#_ENREF_10)**]: comparative efficacy of IFX SC and VDZ in patients with Crohn’s disease.**

| **Treatment period** | **Outcome** | **Group** | **Events** | **Total** | **Proportion (95% CI)** | **Heterogeneity (*I*^2^)** |
| --- | --- | --- | --- | --- | --- | --- |
| Induction | CDAI-70 response | IFX SC^1^ | 42 | 53 | 0.79 (0.66-0.88) | – |
|  |  | VDZ | 500 | 858 | 0.55 (0.37-0.73) | 95% |
|  | CDAI-100 response | IFX SC^1^ | 33 | 53 | 0.62 (0.49-0.74) | – |
|  |  | VDZ | 385 | 1067 | 0.36 (0.33-0.39) | 0% |
|  | Clinical remission | IFX SC^1^ | 26 | 53 | 0.49 (0.36-0.62) | – |
|  |  | VDZ | 209 | 1067 | 0.19 (0.14-0.24) | 77% |
| Maintenance | CDAI-100 response | IFX SC | 186 | 284 | 0.65 (0.60-0.71) | 0% |
|  |  | VDZ | 207 | 423 | 0.48 (0.40-0.57) | 67% |
|  | Clinical remission | IFX SC | 174 | 284 | 0.61 (0.56-0.67) | 0% |
|  |  | VDZ | 189 | 423 | 0.44 (0.34-0.53) | 72% |
| 1 year | Discontinuation due to lack of efficacy | IFX SC | 3 | 53 | 0.05 (0.01-0.28) | – |
|  |  | VDZ | 78 | 275 | 0.33 (0.24-0.42) | 74% |

^1^Results from the induction period were analysed for patients included in the IFX SC group who had received IFX IV induction therapy.

CDAI-70: ≥ 70-point decrease in Crohn’s Disease Activity Index; CI: Confidence interval; IFX: Infliximab; IV: Intravenous; SC: Subcutaneous; VDZ: Vedolizumab.

**Supplementary Table 6 Exploratory analysis for outcomes reported in the LIBERTY-UC study: comparative efficacy of IFX SC and VDZ in patients with ulcerative colitis.**

| **Treatment period** | **Outcome** | **Group** | **Events** | **Total** | **Proportion (95% CI)** | **Heterogeneity (*I*^2^)** |
| --- | --- | --- | --- | --- | --- | --- |
| Maintenance | Clinical response^1,2^ | IFX SC | 206 | 371 | 0.56 (0.50-0.63) | 0% |
|  |  | VDZ | 176 | 282 | 0.63 (0.56-0.69) | 51% |
|  | Clinical remission^1,3^ | IFX SC | 166 | 371 | 0.45 (0.40-0.50) | 0% |
|  |  | VDZ | 243 | 665 | 0.39 (0.33-0.46) | 73% |

^1^Evaluated based on total Mayo score.

^2^For LIBERTY-UC, evaluation was based on decrease in modified Mayo score from baseline (at least 2 points and at least 30%, with an accompanying decrease in the rectal bleeding subscore of at least 1 point or an absolute rectal bleeding subscore of 0 or 1 point).

^3^For LIBERTY-UC, evaluation was based on modified Mayo score (stool frequency subscore of 0 or 1 point, rectal bleeding subscore of 0 point, and endoscopic subscore of 0 or 1 point).

CI: Confidence interval; IFX: Infliximab; SC: Subcutaneous; VDZ: Vedolizumab.

**Supplementary Table 7 Exploratory analysis for outcomes reported in the VISIBLE 2 study [**[**9**](#_ENREF_9)**]: comparative safety of IFX SC and VDZ in patients with Crohn’s disease.**

| **Outcome** | **Group** | **Events** | **Total** | **Proportion (95% CI)** | **Heterogeneity (*I*^2^)** |
| --- | --- | --- | --- | --- | --- |
| AE | IFX SC | 40 | 53 | 0.76 (0.58-0.88) | 0% |
|  | VDZ | 202 | 275 | 0.77 (0.71-0.84) | 80% |
| SAE | IFX SC | 5 | 53 | 0.09 (0.04-0.21) | 0% |
|  | VDZ | 23 | 275 | 0.14 (0.03-0.26) | 97% |
| Infection | IFX SC | 20 | 53 | 0.38 (0.26-0.51) | 0% |
|  | VDZ | 86 | 275 | 0.24 (0.10-0.38) | 94% |
| Serious infection | IFX SC | 2 | 53 | 0.04 (0.01-0.14) | 0% |
|  | VDZ | 4 | 275 | 0.03 (0.00-0.06) | 89% |
| Discontinuation due to AEs | IFX SC | 2 | 53 | 0.04 (0.01-0.14) | 0% |
|  | VDZ | 11 | 275 | 0.07 (0.02-0.12) | 91% |

AE: Adverse event; CI: Confidence interval; IFX: Infliximab; SAE: Serious adverse event; SC: Subcutaneous; VDZ: Vedolizumab.

# REFERENCES

1. Peyrin-Biroulet L, Arkkila P, Armuzzi A, Danese S, Guardiola J, Jahnsen J, Lees C, Louis E, Lukas M, Reinisch W *et al*: **Comparative efficacy and safety of infliximab and vedolizumab therapy in patients with inflammatory bowel disease: a systematic review and meta-analysis**. *BMC Gastroenterol* 2022, **22**(1):291.

2. Schreiber S, Ben-Horin S, Leszczyszyn J, Dudkowiak R, Lahat A, Gawdis-Wojnarska B, Pukitis A, Horynski M, Farkas K, Kierkus J *et al*: **Randomized controlled trial: subcutaneous vs intravenous infliximab CT-P13 maintenance in inflammatory bowel disease**. *Gastroenterology* 2021, **160**(7):2340-2353.

3. **CT-P13 SC trial: unpublished data on file [2023].**

4. Sandborn WJ, Feagan BG, Rutgeerts P, Hanauer S, Colombel JF, Sands BE, Lukas M, Fedorak RN, Lee S, Bressler B *et al*: **Vedolizumab as induction and maintenance therapy for Crohn's disease**. *N Engl J Med* 2013, **369**(8):711-21.

5. Sands BE, Feagan BG, Rutgeerts P, Colombel JF, Sandborn WJ, Sy R, D'Haens G, Ben-Horin S, Xu J, Rosario M *et al*: **Effects of vedolizumab induction therapy for patients with Crohn's disease in whom tumor necrosis factor antagonist treatment failed**. *Gastroenterology* 2014, **147**(3):618-627.e3.

6. Feagan BG, Rutgeerts P, Sands BE, Hanauer S, Colombel JF, Sandborn WJ, Van Assche G, Axler J, Kim HJ, Danese S *et al*: **Vedolizumab as induction and maintenance therapy for ulcerative colitis**. *N Engl J Med* 2013, **369**(8):699-710.

7. Sands BE, Peyrin-Biroulet L, Loftus EV, Jr., Danese S, Colombel JF, Toruner M, Jonaitis L, Abhyankar B, Chen J, Rogers R *et al*: **Vedolizumab versus adalimumab for moderate-to-severe ulcerative colitis**. *N Engl J Med* 2019, **381**(13):1215-1226.

8. Sandborn WJ, Baert F, Danese S, Krznaric Z, Kobayashi T, Yao X, Chen J, Rosario M, Bhatia S, Kisfalvi K *et al*: **Efficacy and safety of vedolizumab subcutaneous formulation in a randomized trial of patients with ulcerative colitis**. *Gastroenterology* 2020, **158**(3):562-572.e12.

9. Vermeire S, D'Haens G, Baert F, Danese S, Kobayashi T, Loftus EV, Bhatia S, Agboton C, Rosario M, Chen C *et al*: **Efficacy and safety of subcutaneous vedolizumab in patients with moderately to severely active crohn's disease: results from the VISIBLE 2 randomised trial**. *J Crohns Colitis* 2022, **16**(1):27-38.

10. Colombel JF, Hanauer SB, Sandborn W, Sands BE, Schreiber S, Danese S, Kierkus J, Kulynych R, Klopocka M, Lahat A *et al*: **DOP86 Subcutaneous infliximab (CT-P13 SC) as maintenance therapy for Crohn’s disease: A phase 3, randomised, placebo-controlled study (LIBERTY-CD)**. *Journal of Crohn's and Colitis* 2023, **17**(1):i161–i162.
